# antiCODE: a natural sense-antisense transcripts database

**DOI:** 10.1186/1471-2105-8-319

**Published:** 2007-08-30

**Authors:** Yifei Yin, Yi Zhao, Jie Wang, Changning Liu, Shuguang Chen, Runsheng Chen, Haitao Zhao

**Affiliations:** 1 Department of Liver Surgery, Peking Union Medical College Hospital, Chinese Academy of Medical Sciences, CAMS & PUMC, Beijing 100730, China; 2 Bioinformatics Group, Institute of Computing Technology, Chinese Academy of Sciences, Beijing 100080, China; 3 Bioinformatics Laboratory, Institute of Biophysics, Chinese Academy of Sciences, Beijing 100101, China; 4 Graduate School of the Chinese Academy of Sciences, Beijing 100080, China

## Abstract

**Background:**

Natural antisense transcripts (NATs) are endogenous RNA molecules that exhibit partial or complete complementarity to other RNAs, and that may contribute to the regulation of molecular functions at various levels. In recent years, large-scale NAT screens in several model organisms have produced much data, but there is no database to assemble all these data. AntiCODE intends to function as an integrated NAT database for this purpose.

**Results:**

This release of antiCODE contains more than 30,000 non-redundant natural sense-antisense transcript pairs from 12 eukaryotic model organisms. In order to provide an integrated NAT research platform, efficient browser, search and Blast functions have been included to enable users to easily access information through parameters such as species, accession number, overlapping patterns, coding potential etc. In addition to the collected information, antiCODE also introduces a simple classification system to facilitate the study of natural antisense transcripts.

**Conclusion:**

Though a few similar databases also dealing with NATs have appeared lately, antiCODE is the most comprehensive among these, comprising almost all currently detected NAT pairs.

## Background

Natural antisense transcripts (NATs) are endogenous RNA molecules that exhibit partial or complete complementarity to other transcripts, through which they may contribute to the regulation of molecular expression at various levels. Though many natural antisense transcripts were discovered through their regulatory function on the expression of mRNAs [1,2], some global predictions of NATs in several species have also been published [3-10]. The first of these used mRNA data to predict natural antisense transcripts [4]. With the appearance of more draft genomes and full length cDNA data, the scale of NATs predictions has been extended. Several datasets, mainly based on full length cDNAs, have been published for mouse [8,11], rice [12] and Arabidopsis thaliana [7]. Since 2006, the trend in NATs prediction has turned to multi-species comparisons [6,13]. A number of published NATs have been validated by various experimental approaches, such as RT-PCR [10] and microarray [5], further confirming that antisense transcript is a common occurrence in eukaryote transcriptomes.

The background for the emergence of so much NAT data in recent years, is on the one hand the availability of more genomic and full length cDNA data, and on the other hand a growing realization of the important functions of natural antisense transcripts. Antisense RNAs may contribute regulatory activity at various levels, such as post-transcription [14,15], splicing [16,17], transport [18], and genomic imprinting[19,20], and have been shown to be involved in the control of developmental processes [21], adaptation to various stresses [22], and viral infection [23,24] through annealing to complementary sequences.

To facilitate research, previous publications have suggested a few classification systems for NATs. The most basic of these is the cis/trans system [4] in which an antisense transcript from the same genomic loci as the sense transcript is labelled a cis-NAT, whereas a trans-NAT is an antisense transcript expressed from a genomic locus different from that of the sense transcript. A second classification system is based on the overlapping position of the complementary pair, which will be divided into 5–6 categories according to their patterns of gene structure, e.g. depending on whether the pair overlaps at their 5' ends, 3' ends, completely, or in the introns [6,7,10,11]. A third classification system considers the respective coding potential of the complementary pair, and includes the categories coding-coding, coding-noncoding and noncoding-noncoding [8,13].

Up to present, a number of large-scale NAT data have been published and several functional studies of NATs have been carried out, however, thus far no database has been set up to collect and order all these transcripts. In order to serve the need of the NAT research, we have over the past two years built the antiCODE database. The purpose of the database is to collect the existing NAT data, and to provide a useful browsing and search platform for these data. This release of antiCODE contains more than 30,000 natural sense-antisense transcript pairs from the 12 model organisms *Homo sapiens *(human), *Mus musculus *(mouse), *Rattus norvegicus *(rat), *Xenopus tropicalis *(western clawed frog), *Drosophila melanogaster *(fruit fly), *Caenorhabditis elegans *(nematode), *Ciona intestinalis *(seasquirt), *Gallus gallus *(chicken), *Danio rerio *(zebrafish), *Bos taurus *(cow), *Oryza sativa *(rice) and *Arabidopsis thaliana *(thale cress).

## Construction and content

All NATs in the database have been collected from recent articles [4-8,10-13]. The original datasets used for construction of the database are listed in Table 1, which include 11,287 human NAT pairs [4,5,10], 14,199 mouse NAT pairs [8,11], 1,339 *A. thaliana *NAT pairs [7], 687 rice NAT pairs [12] and more than 5,000 NAT pairs from other species [6,13].

**Table 1:** The genome-wide NAT datasets in eukaryotic species

Reference	Species involved in the predictions	The number of transcripts
[4]	Human	372
[5]	Human	2,667
[11]	Mouse	4,279
[12]	Rice	1,374
[10]	Human	5,880
[7]	*Arabidopsis thaliana*	1,340
[8]	Mouse	37,562
[13]	Human, mouse, rat, chicken, fruit fly, and nematode	11,200
[6]	Human, mouse, frog, cow, fruit fly, worm, zebra fish and sea squirt	21,266

### Classification

**Figure 1:**
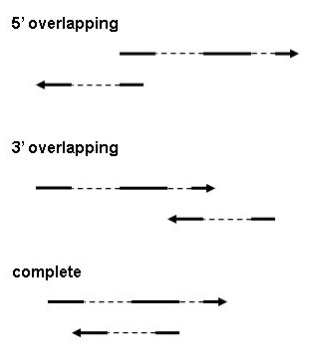
**The "5/3/c/o" classification system**. The arrows indicate the transcriptional orientation of the NAT pair. A solid line indicates an exon and a broken line an intron.

After collecting the NAT pairs, there was a need for uniform criteria to organize the data. Based on the previous classifications, we developed a classification system that includes three complementary aspects for which we use the terms "5/3/c/o", "cis/trans" and "coding/noncoding". The "5/3/c/o" system represents a simplification of the existing classification based on gene structure [6,11], and indicates which parts of the two sequences overlap, i.e. the 5' ends (5' overlapping), the 3' ends (3' overlapping), or one transcript completely covered by the other (complete; see Figure 1). If neither applies, the NAT pair will be marked "o" (other), for instance if only partial overlap between the two transcripts. The "cis/trans" scheme tells whether or not the two sequences of a NAT pair are located at the same chromosomal loci, i.e. if both of them are located at the same genomic position they will be named a cis-NAT pair, otherwise a trans-NAT pair. The "coding/noncoding" scheme indicates whether the two overlapping RNAs are (protein) coding RNAs or noncoding RNAs. We have not adopted the system [6,11] that divided NAT pairs according to their exon-intron structures, because we wish to provide more compact and practical information and thus enable quick retrieval of the most useful bits from the abundance of available information. For more detailed information on particular NAT pairs, users may visit other relevant databases through the provided links.

### Database Construction

We obtained accession numbers and clone IDs for the NAT pairs from the supplementary material of published articles and downloaded the annotation information and sequences from the NCBI and FANTOM websites. In the first step, we divided the NAT pairs to cis/trans classes according to information in referenced papers. The second step was to classify the NAT pairs according to the coding/noncoding system, thus, all NAT pairs were sorted as coding-coding, coding-noncoding and noncoding-noncoding. In the third step, Blat [25] was used to classify the NAT pairs according to the 5/3/c/o system. Finally, we have removed redundant NAT pairs derived from different datasets.

### Website Features

The three core functions of antiCODE database are browse, search and sequence alignment with Blast. Under the browse option, there are five sub-options – Pair ID, cis/trans, overlap, coding/noncoding, and species – by which users can browse all NAT pairs by pair ID, or NAT pair classes.

More specific lookups can be executed by the search function. Users can enter the exact gene accession number or clone ID to see whether a sequence of interest has a possible complementary transcript. If one is interested in NAT pairs relating to some particular condition, e.g. cancer, a relevant key word can be entered in the Text search frame under the search option.

**Figure 2:**
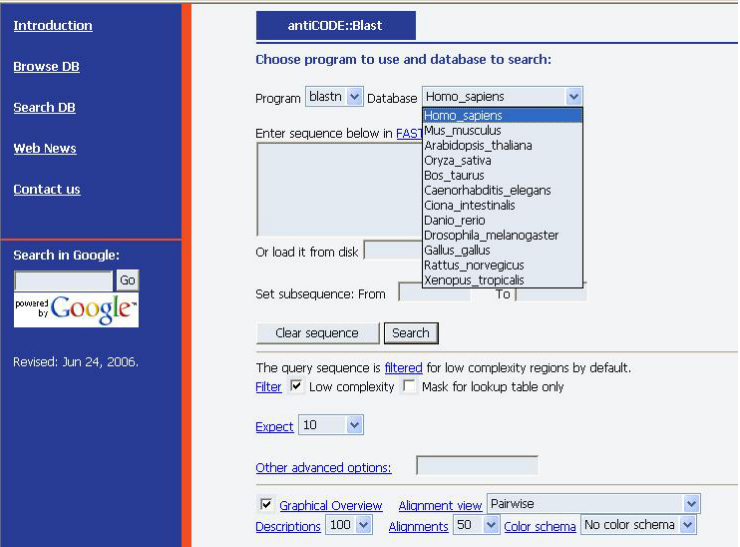
**The Blast options**. In the database frame, 12 genomes could be selected as Blast databases. More detailed options could be found below which allow users to personalize the Blast results according to complexity, expect value and graphical overview options.

If a sequence of interest cannot be found in the database or a user want to investigate whether some novel sequence possibly overlap with known NAT pairs, the Blast option will be very useful. Users just needs to paste her sequence in the sequence window, or load them into the Blast web page, and select the appropriate choices, such as expected number of hits (Figure 2), and then the Blast result will be returned.

After a NAT pairs of interest have been found, all information pertaining to the NAT pair, including annotation and map view links to other databases, affiliated classes, a simple description and references, will appear. More detailed annotations and comments can be obtained through the links to other relevant databases.

## Utility and discussion

Recently, new technologies, such as microarray, SAGE, and MPSS have played prominent roles in the identification of NAT pairs. Before 2005 only EST (UniGene) and mRNAs had been used for NAT prediction. Later large scale full-length cDNA data emerged, based on which more than 1,000 rice NATs[12] were first reported, closely followed by mouse [8,11] and Arabidopsis [7] NATs. For NAT prediction in Arabidopsis [7] also MPSS data has been used, and in 2005, a new NAT dataset based on SAGE was reported in mouse [26]. In 2007, data [27] from whole-genome arrays was employed for NAT prediction in Arabidopsis. It is expected that along with the improvement in array technology, more transcripts from tilling microarrays will be used for future NAT predictions, hopefully resulting in an accurate and exhaustive set of NAT data.

## Conclusion

The most recently released NAT datasets [9,26-28] have yet not been included in antiCODE, but will be included in the next release of the database. However, compared with other existing databases [29], antiCODE is presently the most comprehensive and integrated database for NAT pairs. The most distinctive features of antiCODE are as follows; (i) antiCODE includes almost all known natural antisense transcript (NAT) pairs from 12 eukaryotic model organisms, (ii) antiCODE provides substantial and compact information relating to NATs (e.g. accession number, clone ID, species, classification etc.), (iii) we have introduced a classification system based on the previous notions which should give users an immediate impression of the basic features of each NAT pair, (iv) a Blast service is provided, and (v) antiCODE provides a user-friendly interface and a convenient search option, allowing efficient investigation and verification of natural antisense pairs from different species.

## Availability and requirements

The antiCODE database and related resources can be freely accessed at its websites  or 

## Authors' contributions

Yifei Yin and Yi Zhao carried out the design and the collection of data. Jie Wang carried for building the database. Changning Liu participated in the design of the study. Shuguang Chen helped to draft the manuscript. Runsheng Chen and Haitao Zhao participated in the design and coordination. All authors read and approved the final manuscript.
